# Prevalence of diabetes and other cardiovascular risk factors in an Iranian population with acute coronary syndrome

**DOI:** 10.1186/1475-2840-5-15

**Published:** 2006-07-17

**Authors:** Alireza Esteghamati, Mehrshad Abbasi, Manouchehr Nakhjavani, Abbas Yousefizadeh, Amelita P Basa, Hamid Afshar

**Affiliations:** 1 Associate Professor, Faculty of Medicine, Tehran University of Medical Sciences. Keshavarz Blvd, Tehran, Iran; 2 Endocrine Department, Vali-asr Hospital, Tehran University of Medical Sciences, Keshavarz Blvd, Tehran 14197-33147, Iran; 3 Professor, Internal Medicine, Head of Endocrine Division, Vali-asr Hospital, Tehran University of Medical Sciences, Keshavarz Blvd., Tehran 14197-33147, Iran; 4 Internal Medicine Department, Bank-Melli Hospital, Jomhouri Blvd, Tehran, Iran; 5 Endocrine Dept, VA Medical Center/Baylor College Of Medicine, Houston, TX, USA; 6 Cardiology Dept. Ochsner Clinic Foundation, New orleans, LA, USA

## Abstract

**Background:**

Coronary artery disease is the leading cause of death in industrialized countries and most patients with diabetes die from complications of atherosclerosis. The objective of this study was to determine the presence of diabetes mellitus and other conventional coronary heart disease risk factors (cigarette smoking, hypertension and hyperlipidemia) in patients with acute coronary events in an Iranian population.

**Methods:**

The study included 514 patients with unstable angina or myocardial infarction (MI) out of 720 patients admitted to CCU ward of a general hospital from March 2003 to March 2005. History of diabetes, hypertension and cigarette smoking, demographic indices, coronary heart disease and diabetes mellitus treatment, myocardial enzymes, serum triglycerides (TG) and cholesterol and fasting and non fasting blood glucose levels and HbA1C of diabetics were recorded of admission sheets. The data were structured to appropriate one way ANOVA, T tests, and chi square test with SPSS 13 product for windows.

**Results:**

Out of all patients 35.8% were female, 30% were diabetics (Duration 13.4 ± 8.7 years), 42% were smoker and 91% were hypertensive. Twenty four percent had MI and 76% had unstable angina. MI was significantly higher in diabetic patients (36.4% vs. 19.2%, P < 0.001). Location and extension of MI and myocardial enzymes did not differ between diabetics and non-diabetic patients. Diabetic patients were older than non diabetics (65 ± 11.6 vs. 59.7 ± 12.5 years, p < 0.05). Five (66.7%) out of 9 patients with fatal MI were diabetics (Odds Ratio = 2.98). Age, duration of diabetes and HbA1c levels, did not differ between diabetic patients with or without MI. Hypertension and current smoking was significantly higher in patients with MI compared to patients with unstable angina (p < 0.05). Serum TG, HDL-C, LDL-C and total cholesterol level did not differ between patients with MI and unstable angina. Diabetic patients compare to non diabetic patients were more hypertensive (96% vs. 88.7%, p < 0.005) and had higher serum triglyceride (TG over 200 mg/dl, 35.1% vs. 26.4, p <0.05). Diabetes was more frequent among women than men (36.4% vs. 26.4%, p < 0.05). Women were older than men (65 ± 11.6 vs. 59.2 ± 13 years, p < 0.005) and had higher total serum cholesterol (200 ± 41.8 vs. 192 ± 42.5 mg/dl, p < 0.05) and HDL-C levels (49.7 ± 22 vs. 40 ± 13 mg/dl, p < 0.005). Ninety seven percent of all patients had at least one of cardiovascular risk factors (hypertension, smoking, diabetes, high cholesterol and low HDL-cholesterol levels).

**Conclusion:**

In this study 19 out of 20 patients with acute coronary event have at least one of conventional cardiac risk factors. Diabetes and hypertension are leading risk factors, which may directly or indirectly interfere and predict more serious complications of coronary heart disease.

## Background

The most important complication of Type 2 diabetes Mellitus (DM) is coronary heart disease (CHD) which presents with increased mortality and morbidity compared to the non-diabetic population. In the Framingham Heart Study, the presence of diabetes doubled the age-adjusted risk for cardiovascular disease in men and tripled it in women. Diabetics comprise as many as 20 percent of those who require percutaneous transluminal coronary angioplasty (PTCA) & coronary artery bypass graft surgery (CABG). Coincidence of co-morbidities put diabetic patients in higher risk for CHD and its mortality. Moreover diabetes itself is considered as a CHD risk factor or even CHD equivalent [1]. Patients with diabetes and without previous myocardial infarction (MI) have as high a risk of MI as persons without diabetes and with a previous MI, and that the cardiovascular risk factors of both groups should be treated equally aggressively. Major epidemiological studies raised concerns about the importance of DM as an independent and important CHD risk factor [2]. The aim of this study was to determine the prevalence of diabetes and CHD risk factors in patients with acute coronary syndrome (ACS).

## Methods

We studied the patients who were admitted in CCU ward of a general hospital between March 2003 and March 2005 with ACS. Information was obtained from the archived data sheets about patient demographic characteristics and medical history concerning their age, sex, history of diabetes, duration of diabetes, hypertension, smoking, previous hospital admissions, previous ACS, treatment modalities of coronary heart disease and diabetes, and revascularization. Laboratory data including myocardial enzymes, serum triglycerides and total cholesterol, high-density lipoprotein cholesterol (HDL-C), low-density lipoprotein cholesterol (LDL-C) levels, fasting and non-fasting blood glucose levels (FBS and BS respectively) and glycated hemoglobin (HbA1C) were collected. Diagnosis, type of MI, management and interventional procedures, and in-hospital outcomes were also recorded. The criteria for the diagnosis of MI, type of MI, and unstable angina were based on clinical presentation, biochemical makers of acute ischemic injury, and electrocardiographic findings. Patients were classified as diabetics based on the review of medical records. Dyslipidemia was defined according to ATPIII. Information regarding diabetes treatment modalities (insulin versus oral hypoglycemic agents and/or diet) was also collected.

Five hundred and fourteen out of 720 patients who were admitted to the CCU during interested period were included in the study. Patients with congestive heart failure, cardiomyopathy and severe comorbids and patient who were admitted due to known valvular disease were excluded. The data were structured to appropriate one way ANOVA, T tests, and chi square test with SPSS 13 product for windows

## Results

Out of 514 patients 125 (24.3 %) had MI and 389(75.7%) had unstable angina. Data concerning the prevalence of conventional risk factors in patients with myocardial infarction and unstable angina is shown in table 1. Patients with MI compared to those with unstable angina had higher FBS (158.6 ± 93.6 vs. 116.6 ± 49.9 mg/dl; p < 0.005), BS (189.4 ± 118.4 vs. 139.4 ± 69.3 mg/dl; p < 0.005), and HbA1c (10 ± 2.4 vs. 9.1 ± 3%; p < 0.05). There was no significant difference between patients with MI and unstable angina regarding age (62.2 ± 12.4 vs. 61 ± 13 years) and duration of diabetes (14.5 ± 8.7 vs.12.9 ± 8.7 years). Two third (330, 64.2%) of patients were male. The frequencies of risk factors in different genders are summarized in table 2.

**Table 1:** The prevalence of conventional risk factors in patients with myocardial infarction and unstable angina. * indicates that the difference between patients with UA (unstable angina) and MI (myocardial infarctions) is significant.

	Patients with UA	Patients with MI	All patients
DM*	98(25.2)	56(44.6)	154(30%)
Hypertension+	347(89.2)	120(96)	467(90.9)
Smoking*	150(38.6)	66(52.8)	216(42)
History of previous ACS	51(13.3)	17(13.6)	68(13.3)
Hypertriglyceridemia	200(51.4)	52(41.6)	252(49)
Hypercholesterolemia	157(40.4)	58(46.4)	215(41.8)
High LDL	123(64.1)	34(63)	157(63.8)
Low HDL	150(78.1)	33(61.1)	183(74.4)
total	389(75.7)	125(24.3)	514

**Table 2:** The frequencies of risk factors in different genders. * indicates P < 0.005 and + indicates p < 0.05.

	Female(%)	Male(%)
MI	40(21.7)	85(25.8)
DM+	67(36.4)	87(26.4)
HTN*	179(97.3)	288(87.3)
Smoking*	19(10.3)	197(59.7)
Cholesterol+	199.9+-41.8	192.1+-42.5
Tg	188.4+-88.3	170.6+-97.3
LDL	114+-36	112.3+-37.4
HDL*	49.7+-21.9	40.2+-13.3
Age*	65+-11.6	59.6+-13

Myocardial infarction was more frequent in diabetic patients (one out of three, 36.4%) compared to non-diabetic patients (one out of five, 19.2%, p < 0.005, OR = 2.4). They were more hypertensive than non-diabetic patients (96.1% vs. 88.6, p <0.005) but smoking frequency was the same as non-diabetic patients. Diabetics patients with MI had significantly higher BS (274.7 ± 130.8 vs. 216 ± 89.6 mg/dl, p < 0.005), FBS (222 ± 109.3 vs.167.3 ± 75.6 mg/dl, p <0.01) and HbA1c (10 ± 2.4 vs. 9.1 ± 3%, p < 0.05) than diabetic patients with unstable angina. Comparing diabetic with non-diabetic patients, serum total Cholesterol, LDL-C and HDL-C were the same. There were also the same in hypertensive and normotensive patients.

Diabetic patients had higher serum TG levels than non diabetic patients (179 vs. 161 mg/dl, p < 0.05). The age of diabetic patients with or without MI was the same as non-diabetic patients.

Hypertensive patients had higher MI compared to normotensive patients (25.7% vs.10.6%, p < 0.05). Frequency of MI was higher in smokers than non-smokers (30.6% vs. 19.8%, p <0.005). Smoker patients were younger than non-smoker patients (58.9 ± 13.6 vs. 63 ± 12 years, P < 0.005). Serum HDL-cholesterol level was lower in smoker patients than non-smoker patients (39 ± 10.5 vs. 46.8 ± 20.4 mg/dl, p < 0.005). Accumulation of multiple risk factors in patients with MI and unstable angina is shown in graph 1. Frequency of MI in patients with three risk factors (DM, HTN, and smoking) was significantly higher than patients with no risk factors, one or two risk factors. Frequencies of different types of MI in our patients are listed in table 3.

**Table 3:** Frequencies of different types of myocardial infarction in patients. There were no differences between distribution of MI between those with and without diabetes. Data of 8 patients is missing.

MI distribution	Non diabetics *N*(%)	Diabetics *N*(%)	All Patients *N*(%)
Anterior	19(30)	10(18.9)	29(24.8)
Antrolateral	1(1.6)	2(3.7)	3(2.6)
Antroseptal	8(12.5)	11(20.7)	19(16.2)
Inferior ± Posterior	24(37.5)	17(32.1)	41(35)
Lateral	2(3.1)	1(1.9)	3(2.6)
Septal	0	1(1.9)	1(0.8)
Extensive	1(1.6)	5(9.4)	6(5.1)
Total Q wave	55(86)	47(88.7)	102(87.2)
Non Q wave	9(14)	6(11.3)	15(12.8)

Nine patients died in hospital, which included 5 patients with extensive MI, 2 patients with anterior MI and 2 patients with inferior MI; 5 of them were diabetics (75.6%). BS and HbA1c of patients who died were higher than patients with MI who survived (p < 0.05 and p < 0.005 respectively).

## Discussion

Diabetes, hypertension, smoking and dyslipidemia, are common in patients with ACS. In this study type 2 diabetes was fairy common in patients with acute coronary events as is shown in the San Antonio Heart Study. Unlike other cardiovascular risk factors such as high serum lipid levels, cigarette smoking, and blood pressure, which are either declining or under progressively better medical management and control, and unlike cardiovascular mortality, which is also declining, obesity and type 2 diabetes are exhibiting increasing trends [3].

Moreover CHD is the leading cause of morbidity and mortality in patients with diabetes mellitus and Diabetes is a poor prognostic factor in ACS [4,5]. Our data also shows that patients with diabetes more likely experience acute MI than patients without diabetes in acute coronary events.

Our data concerning higher prevalence of MI and female sex predominance in ACS patients with DM is consistent with report from multinational observational Global Registry of Acute Coronary Events (GRACE),[6]. According to GRACE approximately 1 in 4 patients with ACS presented with a history of diabetes. They were more often women (as in GUSTO-IIb study [7] and Lowel et al [8])and they were also more likely to die during the acute hospitalization with a greater prevalence of comorbidities and coronary risk factors [9]. They were older and less likely to be current cigarette smokers. Other studies showed that diabetic patients with ACS are younger [1,10] and less likely to be current cigarette smoker [11,12]. In this study 1 out of 3 patients with ACS were diabetic and diabetes was more common in females but MI, ACS, and in-hospital mortality frequency were the same in both sexes. Diabetics were older and more hypertensive but as current smoker as non-diabetic patients.

Based on Framingham Study the relative impact of diabetes is substantially greater for women than for men. Whereas non-diabetic women have lower relative CHD risk compared to non-diabetic men, cardiovascular mortality and morbidity was actually about as great for diabetic women as for diabetic men [13] as we found in this study. Although it is suggested that the excess relative risk of CHD mortality in women vs. men with diabetes might disappear after adjusting for classic CHD risk factors [14]

Tight control of BP in diabetic patients reduces all diabetes-related end points and occurrence of stroke, Heart failure and retinopathy but do not interfere with MI, peripheral vascular disease and renal failure [15]. Up to 70% of adult patients with type 2 diabetes have hypertension [16]. The relationship between blood pressure and cardiovascular events in type 2 diabetes is noted in prospective studies [17]. Taken together, these studies suggest that an increase in systolic BP is associated with increased cardiovascular events. This is supported by an epidemiologic analysis of UKPDS data in which each 10 mmHg increase in systolic blood pressure increased the risk of MI by 11%. We also showed that hypertension is common in ACS patients especially in our diabetic patients and it increases the risk of MI.

Serum cholesterol, blood pressure, and smoking are modifiable risk factors and are key points to influence and effect on CHD mortality and risk in diabetes [18]. Dyslipidemia is regarded as highly predictive cardiovascular disease (CVD) risk factor [19] but in our study patients with ACS and DM did not have higher serum cholesterol compared to non-diabetics with ACS. This may be due to treatment of patients with cholesterol lowering agents as shown in GRACE.

We showed that diabetic patients with poor blood glucose control as indicated by higher HbA1c and/or higher BS and FBS had more MI and also non diabetics with elevated blood glucose levels were more likely to have MI. It is possible to simply attribute high FBS/BS to greater stress imposed by MI, but according to analysis of the Paris Prospective study and the British Regional Heart Study, mortality of non-diabetics with isolated 2-h post-challenge hyperglycemia is higher than non-diabetic normoglycemic [20,21]. It is suggested that prediabetic subjects have an atherogenic pattern of risk factors (possibly caused by obesity, hyperglycemia, and especially hyperinsulinemia) which may be present for many years and may contribute to the risk of macrovascular disease [22]. Furthermore in Honolulu Heart Program the age-adjusted and risk factor-adjusted relative risks for CHD, deaths from CHD, and total deaths were higher in the asymptomatic hyperglycemic and known diabetics compared to low- normal euglycemics [23]. On the other side, it is reported that intensive blood glucose control which significantly reduced HbA1c compared to conventional treatment do not reduce significantly risk of diabetes-related death, all-cause mortality, and risk of MI [24]. Hyperinsulinemia and hyperglycemia might affect through their comorbidities as hypertension, dyslipidemia, and central body fat distribution [25,26].

Our study is compatible with previous findings which showed no significant differences in localization of MI and development of Q wave in diabetics and non-diabetic patients [27]

Survival rate after myocardial infarction decreases in type-2 diabetic patient [28], and even diabetes and MI are similarly strong predictors of total mortality [29]. It seems that diabetes independently darkens the prognosis of MI and it is reported that at every risk factor level, the absolute risk of age-adjusted coronary death rate is three times greater for diabetic men than non-diabetic men [30,31]. Our study is concordant with these findings but a limitation of our study was that we only collected the records of in-hospital mortality and based on low mortality rate therefore we can not come to any conclusion about the effect of diabetes on mortality of ACS. Due to our study setting which included patients with relatively regular follow up our results could not be easily generalized to general populations. Furthermore the present study suffers of the limitations of other single center, uncontrolled registry, cross sectional studies, which debilitated us to generalize our results and test the causal association among variables. Finally, we did not have the data concerning central obesity, insulin resistance, and minor and new cardiovascular risk factors including albuminuria, homocystinemia, C-reactive protein (CRP), and adiponectin.

Strength of our study is our Iran population sample, which allows comparison between reportedly encountered data of developed countries and a different ethnicity of a developing country. We would like to highlight the different findings in different genders which might uncover substantial differences between CVD risks males and females.

## Conclusion

Diabetes is common in patients with ACS and is a herald for more severe complications as MI and mortality. This increased risk is partly attributed to hypertension, smoking and dyslipidemia, which are common in patients with ACS. In this study, frequency of hypertension, hypercholesterolemia and smoking in diabetics were the same as non-diabetics, thus diabetes itself play an independent role in increasing CVD risk. Hyperglycemia increases the risk of MI even in non diabetic patients with ACS.

## Competing interests

The author(s) declare that they have no competing interests.

## Authors' contributions

AR conceived and designed the study, carried out the data collection, participated in literature review and edited the manuscript. MA conducted the statistical analyses and literature review, drafted the manuscript, and participated in data collection. MN provided expertise and oversight throughout the process. AY participated in data collection. APB and HA reviewed drafts. All authors read and approved the final manuscript.

**Figure 1:**
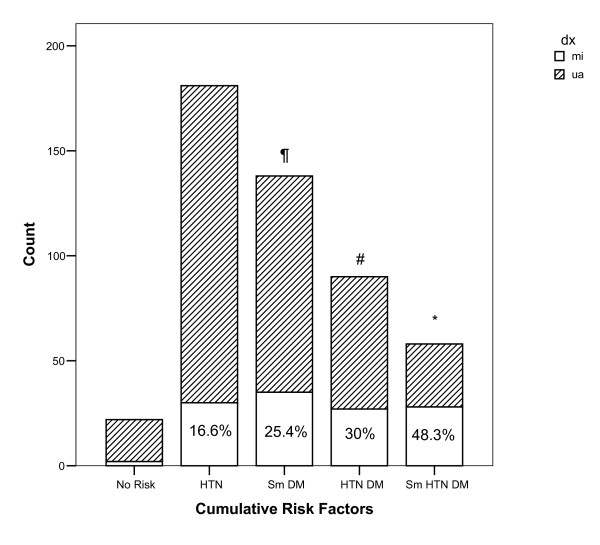
Frequency of myocardial infarction (MI) in patients with multiple risk factors. *indicates significant difference in frequency of MI between patients with smoking (Sm), hypertension (HTN), and diabetes (DM) with other groups. # indicates significant difference in frequency of MI in patients with HTN plus DM compared to patients with HTN and to patients without risk factor. ¶indicates significant difference in frequency of MI between patients with Sm plus DM compare to those with HTN.
